# Is the internet a sufficient source of information on sarcoidosis?

**DOI:** 10.3389/fmed.2023.1217146

**Published:** 2023-06-27

**Authors:** Katharina Buschulte, Philipp Höger, Claudia Ganter, Marlies Wijsenbeek, Nicolas Kahn, Katharina Kriegsmann, Finn M. Wilkens, Jolene H. Fisher, Christopher J. Ryerson, Felix J. F. Herth, Michael Kreuter

**Affiliations:** ^1^Center for Interstitial and Rare Lung Diseases, Pneumology and Respiratory Critical Care Medicine, Thoraxklinik, University of Heidelberg, German Center for Lung Research (DZL), Heidelberg, Germany; ^2^Department of Hematology, Oncology and Rheumatology, Innere Medizin V, Heidelberg University Hospital, Heidelberg, Germany; ^3^Center for Interstitial Lung Diseases and Sarcoidosis, Department of Respiratory Medicine, Erasmus MC-University Medical, Center Rotterdam, Rotterdam, Netherlands; ^4^Laborarztpraxis Rhein-Main MVZ GbR, Limbach Gruppe SE, Frankfurt am Main, Germany; ^5^Department of Medicine, University of Toronto, Toronto, ON, Canada; ^6^Department of Medicine and Centre for Heart Lung Innovation, University of British Columbia, Vancouver, BC, Canada; ^7^Center for Pulmonary Medicine, Departments of Pneumology, Mainz University Medical Center and of Pulmonary, Critical Care and Sleep Medicine, Marienhaus Clinic Mainz, Mainz, Germany

**Keywords:** sarcoidosis, information, internet, quality, content

## Abstract

**Introduction:**

Many patients use the internet as a source of health information. Sarcoidosis is a complex disease, and internet resources have not yet been analyzed for reliability and content on sarcoidosis.

**Aims:**

Our study aimed to investigate the content and the quality of information on sarcoidosis provided by internet resources.

**Methods:**

Google, Yahoo, and Bing were searched for the term “sarcoidosis,” and the first 200 hits were saved in each case. Those websites that met the inclusion criteria (English language, no registration fees, and relevant to sarcoidosis) were then analyzed by two independent investigators for readability, quality (HON, JAMA, and DISCERN), and content (25 predefined key facts) of the provided information.

**Results:**

The websites were most commonly scientific or governmental (*n* = 57, 46%), and the median time since the last update was 24 months. Quality was rated with a median JAMA score of 2 (1; 4) and a median overall DISCERN score of 2.4 (1.1; 4.1), both scores represent partially sufficient information. In total, 15% of websites had a HON certificate. Website content measured by the median key fact score was 19 (ranging from 2.5 to 25) with the lowest scores for acute vs. chronic course of the disease, screening for extrapulmonary disease, and diffuse body pain. Poor results were achieved in industry websites and blogs (*p* = 0.047) with significant differences regarding definition (*p* = 0.004) and evaluation (*p* = 0.021).

**Discussion:**

Sarcoidosis-related content of internet resources is partially sufficient; however, several important aspects are frequently not addressed, and the quality of information is moderate. Future directions should focus on providing reliable and comprehensive information on sarcoidosis; physicians from different disciplines and patients including self-support groups should collaborate on achieving this.

## Introduction

Sarcoidosis is a rare inflammatory granulomatous disease that can affect almost every organ and therefore varies greatly in the course of disease and severity (1). Depending on the organ manifestation, different symptoms can be present, such as dyspnea and cough for pulmonary sarcoidosis, as well as other common symptoms such as fatigue, arthralgia, and body pain (2). It is possible to achieve a cure in many patients. Treatment options are not well established due to a lack of evidence (3), and progression despite treatment occurs in 10–30% of patients with pulmonary sarcoidosis (1). In these severe cases, including the symptom burden and adverse effects of treatment, the patient's quality of life can be substantially impaired (4, 5).

Many patients use internet resources to inform themselves about their health. This is especially true for rare diseases (5) and can lead to a positive impact on quality of life (6). Fisher et al. showed for idiopathic pulmonary fibrosis (IPF), a rare interstitial lung disease, that information on the internet is frequently incomplete and inaccurate (7). Providing adequate information is a particular challenge in sarcoidosis because of the complexity and variability of the disease (8), whilst information on sarcoidosis has been identified as an important need for curing the disease (5).

Our study aimed to evaluate the content and the quality of information on sarcoidosis provided by internet resources.

## Methods

Our study was designed as established by Fisher et al. for IPF (7) and was approved by the Ethics Committee of the Medical Faculty of the University of Heidelberg, Germany (S-435/2021).

### Search strategy and website selection

On 1 August 2021, the three most common search engines (Google, Yahoo, and Bing), which together represent over 96% of the global search market (9), were searched for the term “sarcoidosis.” Before the search was performed, all cookies and histories of web browsers were deleted. This process was repeated individually for the three search engines, and the first 200 hits were saved in each case.

The websites and first generation links within the same domain were then screened systematically for inclusion by one author (KB). Exclusion criteria included duplicates and non-English websites, those requiring registration or enrolment fees, websites not relevant to sarcoidosis, and those that had a clear scientific motive but lacked patient-related focus.

### Data extraction and website evaluation

General information about the included websites was collected, including URL, search rank, host country and continent, and most recent update and sponsoring (advertisement). Each website was assigned to one of the following five categories: scientific/governmental, foundation/advocacy, news/media, industry/for profit, and personal commentary/blog. In addition, it was recorded whether a Health on the Net (HON) code certification existed. The HON code certification has been created for websites offering health information and is provided by an independent organization (10). The Flesch Reading Ease Score [FRES, (11)] and Flesch Kincaid Grade Level [FKGL, (12)] were determined for all website text.

Subsequently, each website and the first-generation links were analyzed thoroughly for content. We defined 25 key facts on sarcoidosis based on current guidelines (2, 13, 14) together with experts in this field (MW, NK, and MK) and decided whether they were fully addressed (1 point), partially addressed (0.5 points), or not addressed (0 points). The key facts were sub-grouped into different categories such as definition, symptoms, risk factors, evaluation, management, and outcome. Finally, wrong or misleading facts were also listed.

For quality analysis of information, we used the DISCERN instrument (15) and *Journal of the American Medical Association* (JAMA) dichotomous benchmarks considering the items such as authorship, attribution, disclosure, and currency (Supplementary material S1) (16). The DISCERN instrument is a validated questionnaire that can be applied to any disease to assess the quality of patient information. DISCERN consists of 16 questions, including 8 on reliability, 7 on specific details regarding treatment choices, and 1 on overall quality. Each question is scored from 1 (quality criterion has not been fulfilled) to 5 (completely fulfilled; Supplementary material S2) (15).

The results were compared after each website had been evaluated independently by two experienced investigators (KB and PH). Re-evaluation of the websites was followed by a discussion between the two reviewers in the case of remaining disagreements, defined as initial scores differing by more than 1 point (DISCERN) or more than 0.5 points (key facts).

### Statistical analysis

Statistical data evaluation was performed in a descriptive manner and is provided as absolute numbers (percentages), median [minimum; maximums], and mean [standard deviation (SD)]. Between-group differences of websites by category were analyzed by the Kruskal–Wallis test. The Mann–Whitney U-test and unpaired *t*-test were used to test for inter-group comparisons depending on the availability of the HON foundation certificate. Statistical significance was set by a two-sided p-value of < 0.05. No adjustment was made for multiple testing. Data analysis was performed using Excel and RStudio 2022.12.0.

## Results

### Website characteristics

**Figure 1:**
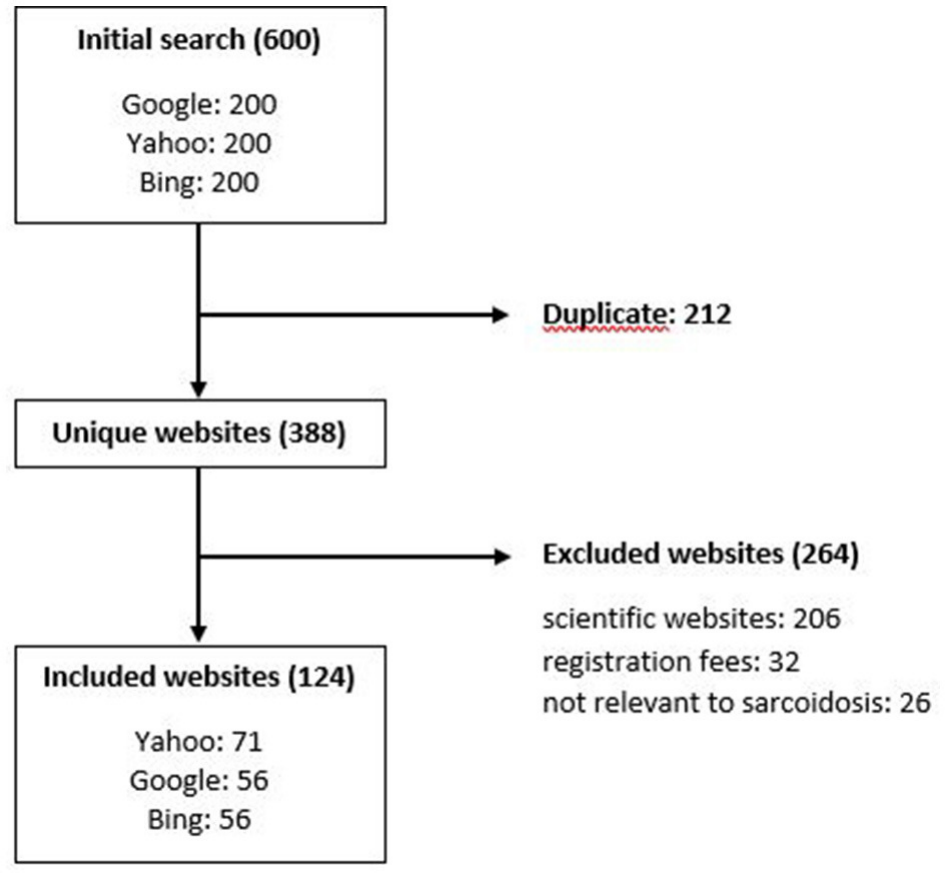
Search results and selection for English websites. Each included website was assigned to all search engines where hits were registered.

The first 200 hits from Google, Yahoo, and Bing included 212 duplicates. Subsequently, 206 websites were excluded because they were not directed at a patient audience (scientific websites), 32 due to requiring registration fees, and 26 for not being relevant to sarcoidosis. The final analysis included 124 unique websites (Figure 1), as listed in the (Supplementary material S3).

The characteristics of the included websites are listed in Table 1. Most websites were included from Yahoo (*n* = 71, 57.3%) and 56 websites each of Google and Bing (45.2% each). Scientific or governmental websites were the most common website type (*n* = 57; 46%), followed by 30% news/media (*n* = 37). Advertising was present on 35% of websites. The majority of websites did not have a certification from the HON foundation (*n* = 106, 85%). The Median JAMA score was 2 (1; 4), representing partially sufficient information. The median overall DISCERN score was 2.4 (1.1; 4.1).

**Table 1:** Characterization of unique websites.

**Overall unique websites**, ***n*** **(%)**			**124 (100)**
General information	Website category, *n* (%)	Scientific/Governmental	57 (46)
		Foundation/Advocacy	16 (13)
		News/media	37 (30)
		Industry/for-profit organization	9 (7)
		Personal commentary/blog	5 (4)
	Host continent, *n* (%)	Europe	19 (15)
		North America	95 (77)
		South America	0 (0)
		Asia	3 (2)
		Australia	7 (6)
		Africa	0 (0)
		Antarctica	0 (0)
	Sponsored websites, *n* (%)	Yes	44 (35)
		No	80 (65)
General quality of medical information	HON foundation certificate	Yes (%)	18 (15)
		No (%)	106 (85)
	JAMA score	Median (range)	2 (1–4)
Patient- focused information	Sum DISCERN score	Median (range)	2.4 (1.1–4.1)
	Readability	Flesch Reading Ease Score, Mean (SD)	42 (13)
		Flesch Kincaid Grade Level, Mean (SD)	9 (2)
	Specific entity-related content	Sum key fact score, Median (range)	19 (2.5–25)

HON, Health on the Net; JAMA, Journal of the American Medical Association; SD, standard deviation.

Readability was analyzed by the Flesch Reading Ease Score (42, SD 13) and Flesch Kincaid Grade Level (9, SD 2). The scores corresponded to the best understanding by the college or (FRES) university graduates (FKGL) and thus stand for difficult readability. There were no differences in readability between different website categories (Supplementary material S4).

**Figure 2:**
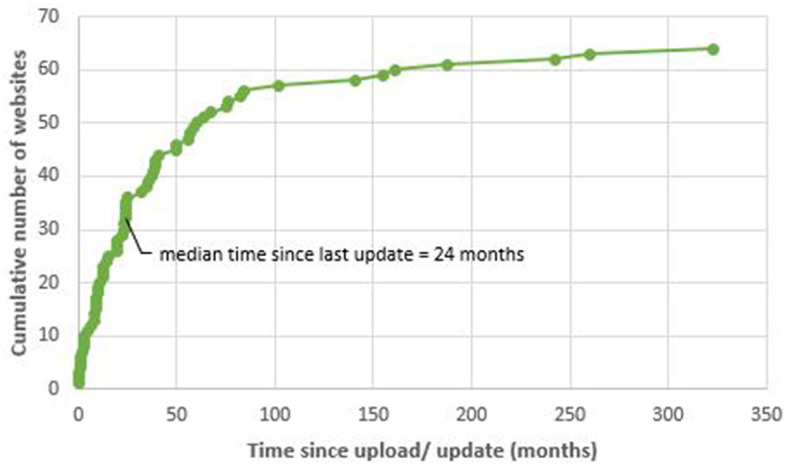
Time since website upload. The plot shows the websites ordered by their time since upload/update until the date of assessment in months. Only websites with available publishing/update dates were included (*n* = 64).

Median time since the last update was 24 months (0–323) for the 64 websites (52%) that reported this information (Figure 2). Foundation/advocacy websites tended to be more current (median 17 months, ranging from 13 to 82) in comparison to industry websites (median 102 months) and blogs (median 70 months, ranging from 1 to 84; Supplementary material S4).

### Website content

**Figure 3:**
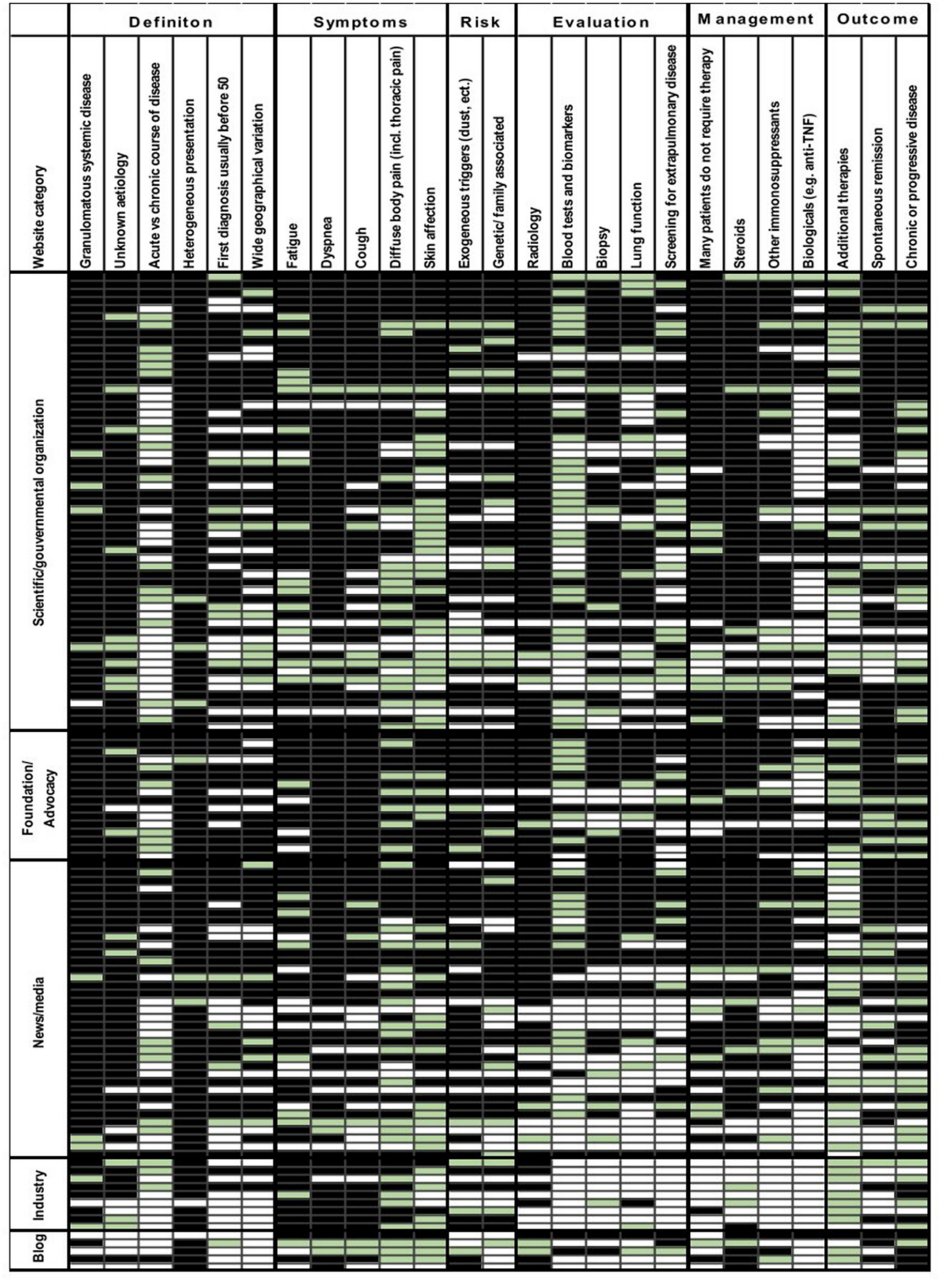
Key fact scores for English websites. Key fact items (columns, *n* = 25) are shown for single websites (rows, *n* = 124). The categorial key fact item scoring is 0 (not addressed), 0.5 (partially addressed), and 1 (fully addressed). The websites are grouped by website category. ACE, angiotensin-converting enzyme; IL-2, interleukine 2; anti-TNF, anti-tumor necrosis factor. 0: white, 0.5: green, and 1: black.

The median key fact score was 19 (ranging from 2.5 to 25), indicating that most websites addressed a majority of the content thought to be relevant to patients. The evaluation of website content revealed relevant differences with regard to different key facts and website categories (Figure 3). The definition of sarcoidosis was mostly well explained, e.g., sarcoidosis as a granulomatous systemic disease (89%) and heterogeneous presentation with various organ involvement (94%). However, the differentiation between the acute and chronic course of the disease was not mentioned in 47% of the websites and only partially discussed in 24%. Common symptoms of the disease including dyspnea (85%) and cough (73%) were often fully addressed. Other symptoms including fatigue (64%), diffuse body pain (50%), and skin involvement (48%) were less frequently explained. Most websites discussed key components of the diagnostic evaluation, including radiology (78%) and biopsy (60%). In contrast, 41% of the websites contained no information and 19% had partial information on screening for extrapulmonary disease. Information on lung function (38%) and blood tests and biomarkers (40%) was seldom presented. The role of corticosteroids (79%) and the findings that many patients do not require therapy (69%) were often stated; in contrast, biologicals (32%) and additional therapies (30%) were rarely mentioned. Information on risk factors and outcomes was provided on 59–66% of the websites.

Wrong or misleading facts were presented on 15 websites (12%), which mostly concerned the therapy of sarcoidosis. Corticosteroids were either presented as absolutely necessary or with a long therapy duration of 12 months or more in contrast to the recent ERS clinical practice guidelines (2). One website listed corticosteroids as the only treatment option and another stated that no treatment is necessary for cardiac sarcoidosis.

Comparison between the different website categories showed the highest scores (i.e., best content) for scientific/governmental, foundation/advocacy, and news/media websites. In contrast, poor results were observed for industry websites and blogs (*p* = 0.047). Significant differences across sites were found with respect to definition (*p* = 0.004) and evaluation (*p* = 0.021). Very few industry websites and blogs contained information on evaluation and management.

Finally, the websites were examined for a possible association between search rank and content. Therefore, websites were sorted by a primary criterion “sum key fact score.” There was no clear association between search rank and content.

### Website quality

**Figure 4:**
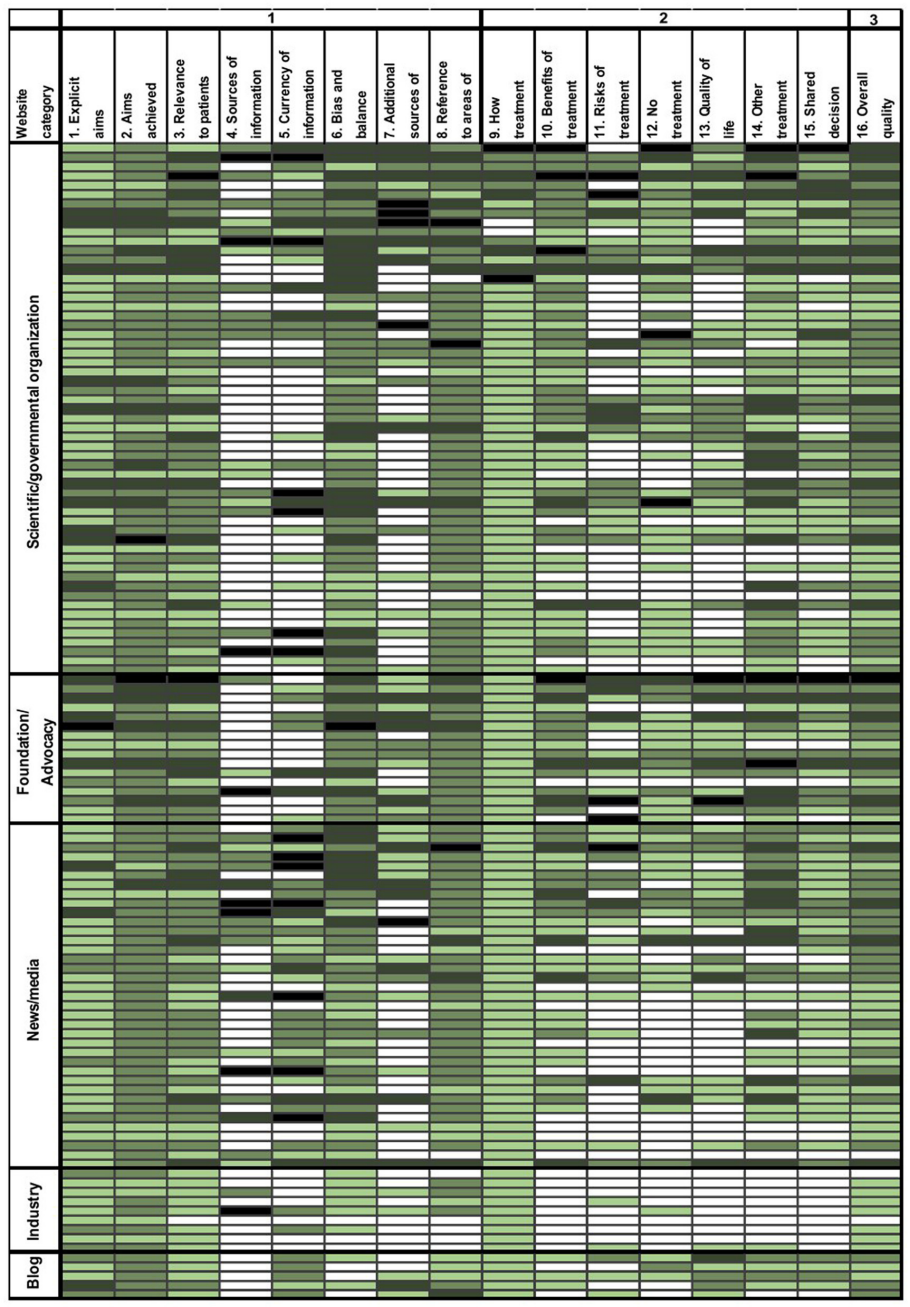
DISCERN scores grouped by website category. DISCERN score items (columns, *n* = 16) are shown for single websites (rows, *n* = 124). The categorial DISCERN item scoring ranges between 1 (not addressed) and 5 (fully addressed). 1: white, 2: light green, 3: green, 4: dark green, and 5: black.

Website quality was measured with the DISCERN instrument and yielded values in a medium range (3 points, range 1–5). Based on all rated websites, poor results were achieved for the questions on sources of information (1 point, range 1–5), currency of information (2 points, range 1–5), and additional sources of information (2 points, range 1–5). Regarding the quality of information on treatment choices (Section Methods of DISCERN), almost all categories were barely fulfilled with a median score of 2 points. Risks of treatment were rarely mentioned (1.5 points, range 1–5; Figure 4).

Foundation and advocacy websites reached the highest DISCERN scores in all three sections (Table 2). In contrast, industry websites achieved poor results (*p* < 0.001). This was particularly evident for items 9–15 regarding the quality of information on treatment choices (*p* < 0.001).

**Table 2:** Sum DISCERN scores for different sections by the website category.

	**Scientific/ governmental**	**Foundation/ advocacy**	**News/media**	**Industry/ for profit**	**Personal commentary/blog**	***p*-value**
Section 1 (items 1–8): Is the publication reliable? median (range)	2.6 (1.8–4)	2.8 (2–3.8)	2.5 (1.6–3.5)	1.8 (1.3–2.8)	2.1 (1.9–2.8)	0.002
Section 2 (items 9–15): How good is the quality of information on treatment choices? median (range)	2.1 (1–4.3)	2.6 (1–4.7)	1.9 (1–3.7)	1 (1–1.4)	1.9 (1.3–2.7)	<0.001
Section 3 (item 16): Overall rating of the publication median (range)	3 (2–4)	3 (2–5)	3 (1–4)	2 (1–2)	3 (2–3)	0.002
Overall median (range)	2.5 (1.4–3.8)	2.9 (1.6–4.1)	2.4 (1.4–3.5)	1.5 (1.1–2)	2.2 (1.8–2.4)	<0.001

The JAMA score differed between website categories with the highest median score for news/media (3, range 1) and poor results for industry websites (1, range 1; *p* < 0.001; Supplementary material S4). Certification from the HON foundation was present in 7% of scientific websites (*n* = 4) and 38% of news/media (*n* = 14). Websites with HON certification had higher JAMA scores (*p* < 0.001) and better results in summed DISCERN score (*p* = 0.003). In addition, they tended to have better evaluations of content measured by summed key fact score (*p* = 0.015). No correlation was found between HON certification and readability (FRES *p* = 0.349 and FKGL *p* = 0.682).

In addition, the websites were sorted by search rank and a secondary criterion “DISCERN sum overall,” and no clear association was found between the search rank and the quality of the websites.

## Discussion

Within the framework of this study, we investigated the content and the quality of internet resources on sarcoidosis. Therefore, 124 eligible English websites were systematically evaluated with different validated instruments. Sarcoidosis-related content of internet resources showed to be partially sufficient. However, several important aspects are frequently not addressed, and the quality of information is moderate. This is highly relevant because the internet presents a common source of health information to patients. In a German survey-based study, 94% of patients with sarcoidosis used the internet to obtain information on their disease (17). To the best of our knowledge, this has not been studied to date.

Most of the websites that met the inclusion criteria were scientific/governmental websites. In a comparison analysis of IPF, the largest group was also scientific/governmental (7). Just as with IPF, most eligible websites were found on Yahoo (7). Websites in our analyses were frequently not up to date with a median time since the last update of 24 months indicating that the information provided on these sites may not reflect the current status of guideline recommendations, especially regarding new therapies such as biologics (2). Information on industry websites and blogs was particularly outdated, but in comparison, foundation/advocacy websites were best updated.

In general, there was greater quality content provided on scientific/governmental websites, and less content on news/media websites, which were particularly lacking details on evaluation and management. Consistent with our results, scientific websites on IPF similarly provided the most content (7) as did Youtube videos on lymphangioleiomyomatosis (LAM) (18).

Overall, website content measured by predefined key facts was acceptable with a median of 19 out of 25 points. However, several aspects were not addressed regardless of the website category. These included the acute vs. chronic course of the disease, screening for extrapulmonary disease, and the common symptom of diffuse body pain. These findings are consistent with a German survey, where relevant information gaps included fatigue and diffuse pain as well as the different courses of disease (17). Additionally, information gaps included the management of sarcoidosis and were further accentuated by reporting of wrong or misleading facts that primarily concerned false information regarding the necessity and duration of corticosteroid therapy as well as treatment indications. Only one-third of the websites provided adequate information on biologics and additional therapies such as rehabilitation. Patients with sarcoidosis often want to be involved in treatment decisions in terms of a shared decision-making process (19), which can lead to better outcomes and treatment adherence (8). However, in a Dutch study, 57% of sarcoidosis patients stated that they cannot find sufficient information about their disease (19); one of the reasons for this is likely the heterogeneity and complexity of sarcoidosis (8). Even with other diseases, such as cancer, the information needed for participating in shared decision-making is often not available on the internet (20). Our results have highlighted that information on the management and therapy of sarcoidosis is often missing or misleading. This poses challenges for all parties involved. If the information available on the internet differs from that of the attending physician, this can lead to negative interactions and disruptions in doctor–patient relationships (21).

In addition to often missing relevant content, the quality, reliability, and readability were also found to be moderate to poor in our analyses. Readability corresponded to college or university graduates, which is far beyond what is recommended for health information disseminated to a patient audience, e.g., the National Institutes of Health (NIH) recommend a grade of 6–7 reading level meaning that comprehension should be easy or fairly easy to read (22). The median DISCERN score of 2.4 points and the median JAMA score of 2 points also shows that the quality of websites is not sufficient, particularly with respect to the currency of information and information on treatment choices (especially risks of treatment). In addition, very poor scores regarding sources of information are another important factor, although citing sources is essential in terms of reliability. In comparison to other diseases, the quality of internet resources measured by DISCERN and JAMA was also poor for IPF (7) and breast cancer (20), while in contrast, information on prostate cancer achieved good results (23). The quality of online health information, in general, has been studied in several meta-analyses (24); until 2002, 70% of the analyzed studies stated that the quality was a problem (25), and from 2002 to 2013, the quality of online health information was found to be problematic in 55% of the analyzed studies (26). This lack of reliability poses a special challenge to patients who may have greater difficulty recognizing risk and bias in online information (7). Thus, 63.4% of sarcoidosis patients reported the internet to be a reliable source of information despite the limitations of this source (17).

This study illustrates several problems in internet resources on sarcoidosis. It should be emphasized that quality was often insufficient, and information was lacking concerning some very relevant aspects, especially with regard to therapy. Therefore, we require better guidance and methods for patients, where they can obtain information about their disease. One possible tool could be HON certification as this resulted in better DISCERN and JAMA scores and tended to be connected to better content.

This study has some limitations. We analyzed websites in English only. Therefore, our results are mainly helpful to patients who use English-language information on the internet. Content and quality of internet resources on sarcoidosis may vary in other languages. In addition, only the largest three platforms, namely Google, Yahoo, and Bing, were searched. We performed our search on a single date; therefore, we did not consider changes in content over time. Furthermore, we have only searched for the term “sarcoidosis,” possible abbreviations as well as different terms for the disease, e.g., Löfgren syndrome, have been disregarded. Websites with registration fees were not included in our analysis; we cannot exclude that these websites may not have a higher content quality than the free-to-use sites reported here. The content analysis was based on previously defined key facts by various sarcoidosis experts, which are therefore not validated. Demographic data of the website readers are lacking. Thus, it is not possible to find a correlation between the readers and website categories. Despite these limitations, we have identified important gaps in the quality of online patient-directed sarcoidosis health information.

## Conclusion

Our results clarify that the content and quality of internet resources on sarcoidosis are acceptable but with several important aspects that are frequently not addressed. In order to facilitate shared decision-making, efforts should be directed toward obtaining reliable and comprehensible information, especially due to the complexity of the disease and the increase in different treatment options. For this purpose, physicians from different disciplines and patients including self-support groups should collaborate together.

## Data availability statement

The original contributions presented in the study are included in the article/Supplementary material, further inquiries can be directed to the corresponding author.

## Ethics statement

The study was approved by the Ethics Committee of the Medical Faculty of the University of Heidelberg, Germany (S-435/2021).

## Author contributions

KB, CG, and MK were responsible for the study design and this was adapted to a study in IPF by CR and JF. The key facts were developed by KB, MW, NK, and MK. Website evaluation was performed by KB and PH. KK was responsible for the statistical analyses. KB was a major contributor in writing the manuscript. FW and FH had contributions to the conception of the work and for the preparation of the final manuscript. All authors read and approved the final manuscript.
