# Supplementary material for: Is the internet a sufficient source of information on sarcoidosis?
Source: Front Med (Lausanne). 2023 Jun 27;10:1217146. doi: 10.3389/fmed.2023.1217146 (PMC10333509; doi:10.3389/fmed.2023.1217146)
Supplement: Supplementary file 1 [file Table_1.DOCX]

**Supplement**

**Supplement S1** JAMA score. Each item is scored with zero (not fulfilled) or one point (fulfilled) and a sum score is calculated (0 – 4 points) [15].

| **Item** | **Description** | **Score** |
| --- | --- | --- |
| Authorship | Authors and contributors, their affiliations, and relevant credentials should be provided | 1 |
| Attribution | References and sources for all content should be listed clearly, and all relevant copyright information noted. | 1 |
| Disclosure | Website"ownership" should be prominently and fully disclosed, as should any sponsorship, advertising, underwriting, commercial funding arrangements or support, or potential conflicts of interest. | 1 |
| Currency | Dates that content was posted and updated should be indicated. | 1 |

**Supplement S2** DISCERN instrument. Scoring from 1 (quality criterion has not been fulfilled) to 5 (completely fulfilled) [14].

| **Section** | **Question** | **Score** |
| --- | --- | --- |
| **Section 1**  **–**  **Reliability** | Q1: Are the aims clear? | 1-5 |
|  | Q2: Does it achieve its aims? | 1-5 |
|  | Q3: Is it relevant? | 1-5 |
|  | Q4: Is it clear what sources of information were used to compile the publication (other than the author or producer)? | 1-5 |
|  | Q5: Is it clear when the information used or reported in the publication was produced? | 1-5 |
|  | Q6: Is it balanced and unbiased? | 1-5 |
|  | Q7: Does it provide details of additional sources of support and information? | 1-5 |
|  | Q8: Does it refer to areas of uncertainty? | 1-5 |
| **Section 2**  **–**  **Treatment choices** | Q9: Does it describe how each treatment works? | 1-5 |
|  | Q10: Does it describe the benefits of each treatment? | 1-5 |
|  | Q11: Does it describe the risks of each treatment? | 1-5 |
|  | Q12: Does it describe what would happen if no treatment is used? | 1-5 |
|  | Q13: Does it describe how the treatment choices affect overall quality of life? | 1-5 |
|  | Q14: Is it clear that there may be more than one possible treatment choice? | 1-5 |
|  | Q15: Does it provide support for shared decision-making? | 1-5 |
| **Section 3**  **–**  **overall rating** | Q16: Based on the answers to all of the above questions, rate the overall quality of the publication as a source of information about treatment choices. | 1-5 |

**Supplement S3** List of included websites with search ranks. In the case of several specified search engines, the search rank refers in each case to the first of the named search engines.

| **Search engine** | **Website URL** | **Search rank** |
| --- | --- | --- |
| Google, Bing, Yahoo | https://www.mayoclinic.org/diseases-conditions/sarcoidosis/symptoms-causes/syc-20350358 | 1 |
| Google, Bing, Yahoo | <https://en.wikipedia.org/wiki/Sarcoidosis> | 3 |
| Google, Bing | <https://www.nhs.uk/conditions/sarcoidosis/> | 4 |
| Google, Bing, Yahoo | <https://www.webmd.com/lung/arthritis-sarcoidosis> | 5 |
| Google, Bing, Yahoo | <https://www.healthline.com/health/sarcoidosis> | 6 |
| Google, Bing, Yahoo | [https://my.clevelandclinic.org/health/diseases/11863-sarcoidosis-overview](https://emedicine.medscape.com/article/301914-overview) | 7 |
| Google, Bing, Yahoo | <https://www.stopsarcoidosis.org/what-is-sarcoidosis/> | 9 |
| Google, Bing | <https://www.blf.org.uk/support-for-you/sarcoidosis/what-is-it> | 10 |
| Google, Yahoo | <https://www.hopkinsmedicine.org/health/conditions-and-diseases/pulmonary-sarcoidosis> | 11 |
| Google, Bing, Yahoo | <https://www.nhlbi.nih.gov/health-topics/sarcoidosis> | 12 |
| Google, Bing | <https://medlineplus.gov/sarcoidosis.html> | 14 |
| Google, Yahoo | <https://www.lung.org/lung-health-diseases/lung-disease-lookup/sarcoidosis/symptoms-diagnosis> | 15 |
| Google | <https://www.orpha.net/consor/cgi-bin/OC_Exp.php?Lng=GB&Expert=797> | 16 |
| Google | <https://dermnetnz.org/topics/sarcoidosis/> | 17 |
| Google, Bing | <https://www.betterhealth.vic.gov.au/health/conditionsandtreatments/sarcoidosis> | 18 |
| Google, Yahoo | <https://www.medicinenet.com/sarcoidosis/article.htm> | 23 |
| Google, Bing, Yahoo | <https://rarediseases.info.nih.gov/diseases/7607/sarcoidosis> | 26 |
| Google, Yahoo | <https://www.cedars-sinai.org/health-library/diseases-and-conditions/p/pulmonary-sarcoidosis.html> | 27 |
| Google, Bing, Yahoo | <https://www.msdmanuals.com/professional/pulmonary-disorders/sarcoidosis/sarcoidosis> | 30 |
| Google, Yahoo | <https://foundation.chestnet.org/lung-health-a-z/sarcoidosis/> | 36 |
| Google, Yahoo | <https://www.verywellhealth.com/sarcoidosis-symptoms-treatment-2861011> | 39 |
| Google, Bing, Yahoo | <https://www.medicalnewstoday.com/articles/318105> | 41 |
| Google, Yahoo | <https://www.thoracic.org/patients/patient-resources/resources/what-is-sarcoidosis.pdf> | 48 |
| Google | <https://www.pulmonologyadvisor.com/home/decision-support-in-medicine/pulmonary-medicine/sarcoidosis-4/> | 50 |
| Google, Yahoo | <https://www.ucsfhealth.org/conditions/sarcoidosis> | 78 |
| Google, Bing, Yahoo | <https://www.mountsinai.org/health-library/diseases-conditions/sarcoidosis> | 80 |
| Google | <https://www.sarcoidosisuk.org/information-hub/about-sarcoidosis/> | 83 |
| Google, Yahoo | <https://www.ottawaheart.ca/heart-condition/cardiac-sarcoidosis> | 84 |
| Google | <https://www.uofmhealth.org/conditions-treatments/pulmonary/sarcoidosis> | 85 |
| Google | <https://www.pennmedicine.org/for-patients-and-visitors/patient-information/conditions-treated-a-to-z/sarcoidosis> | 86 |
| Google, Bing | <https://www.healthdirect.gov.au/sarcoidosis> | 87 |
| Google, Bing | <https://familydoctor.org/condition/sarcoidosis/> | 89 |
| Google | <http://www.idph.state.il.us/public/hb/hbsarcoi.htm> | 97 |
| Google | <https://www.med.unc.edu/medicine/pulmonary/patient-care/specialties/sarcoidosis/> | 99 |
| Google | <https://www.aad.org/public/diseases/a-z/sarcoidosis-treatment> | 104 |
| Google | <https://uvahealth.com/services/pulmonary/sarcoidosis> | 105 |
| Google, Bing, Yahoo | <https://sarcoidosisnews.com/> | 108 |
| Google, Bing, Yahoo | <https://www.physio-pedia.com/Sarcoidosis> | 109 |
| Google, Yahoo | <https://www.brighamandwomens.org/lung-center/diseases-and-conditions/sarcoidosis> | 113 |
| Google | <https://www.healthnavigator.org.nz/health-a-z/s/sarcoidosis/> | 118 |
| Google, Bing | <https://lungfoundation.com.au/wp-content/uploads/2018/09/Factsheet-Sarcoidosis-Jul2016.pdf> | 120 |
| Google | <https://pulmonary.ucsf.edu/care/sarcoidosis> | 122 |
| Google | <https://sleepandlungcare.com.au/sarcoidosis/> | 125 |
| Google, Yahoo | <https://nyulangone.org/conditions/sarcoidosis-in-adults> | 128 |
| Google, Bing | <https://www.rbht.nhs.uk/our-services/sarcoidosis> | 135 |
| Google | <https://www.dph.illinois.gov/topics-services/diseases-and-conditions/diseases-a-z-list/sarcoidosis> | 139 |
| Google | <https://www.nationaljewish.org/NJH/media/pdf/pdf-MF-Cardiac-Sarcoidosis.pdf?modal=1> | 141 |
| Google | <https://www.rileychildrens.org/health-info/sarcoidosis> | 143 |
| Google, Bing, Yahoo | <https://www.nm.org/conditions-and-care-areas/pulmonary/sarcoidosis> | 148 |
| Google | <https://www.actionpf.org/information-support/what-is-sarcoidosis> | 166 |
| Google | <https://www.bmc.org/sarcoidosis/about-sarcoidosis> | 169 |
| Google, Yahoo | <https://wexnermedical.osu.edu/lung-pulmonary/sarcoidosis> | 178 |
| Google | <https://www.henryford.com/services/sarcoidosis> | 179 |
| Google | <https://www1.nyc.gov/assets/911health/downloads/pdf/wtc/SarcoidosisFS.pdf> | 188 |
| Google | <https://www.healthlinkbc.ca/health-topics/abl2958> | 193 |
| Bing | <https://www.netdoktor.de/krankheiten/sarkoidose> | 7 |
| Bing, Yahoo | <https://www.wikihow.com/Deal-With-Sarcoidosis> | 20 |
| Bing | <https://thecurbsiders.com/podcast/256> | 31 |
| Bing, Yahoo | <https://www.news-medical.net/health/What-is-Sarcoidosis.aspx> | 55 |
| Bing | <https://myhealth.alberta.ca/health/Pages/conditions.aspx?hwid=abl2958> | 65 |
| Bing, Yahoo | <http://www.umsarcoidosis.com/> | 91 |
| Bing | <http://www.health-reports.com/sarcoidosis.shtml> | 93 |
| Bing | <https://www.emedicinehealth.com/life_expectancy_of_a_person_with_sarcoidosis/article_em.htm> | 98 |
| Bing, Yahoo | <https://strongerthansarcoidosis.org/> | 99 |
| Bing | <https://www.lung.ca/lung-health/lung-disease/sarcoidosis/treatment> | 103 |
| Bing | [https://www.bad.org.uk/shared/get-file.ashx?id=230&itemtype=document - British Association of ... Sarcoidosis - Health Reports](https://www.bad.org.uk/shared/get-file.ashx?id=230&itemtype=document%20-%20British%20Association%20of%20...%20Sarcoidosis%20-%20Health%20Reports) | 104 |
| Bing | <https://www.planetayurveda.com/ayurvedic-treatment-for-sarcoidosis/> | 111 |
| Bing, Yahoo | <https://healthjade.com/sarcoidosis/> | 116 |
| Bing | <https://www.reddit.com/r/sarcoidosis/> | 121 |
| Bing | <https://flexikon.doccheck.com/en/Sarcoidosis> | 122 |
| Bing | <https://berniemacfoundation.org/about-sarcoidosis/> | 123 |
| Bing | <https://www.europeanlunginfo.org/sarcoidosis/> | 125 |
| Bing | <https://www.britannica.com/science/sarcoidosis> | 126 |
| Bing | <https://abcnews.go.com/Health/story?id=5589149&page=1> | 131 |
| Bing | <https://ccohs.ca/oshanswers/diseases/sarcoido.html> | 132 |
| Bing | <https://www.medindia.net/patients/patientinfo/sarcoidosis.htm> | 138 |
| Bing | <https://www.thefreelibrary.com/Sarcoidosis.-a017246670> | 140 |
| Bing, Yahoo | <https://mpkb.org/home/diseases/sarcoidosis> | 142 |
| Bing, Yahoo | <https://pulmonaryhypertensionnews.com/pulmonary-hypertension-sarcoidosis/> | 149 |
| Bing, Yahoo | <https://www.upmc.com/services/pulmonology/conditions/sarcoidosis> | 153 |
| Bing | <https://www.sarcoidosisri.org/complementary-medicine/mental-health/> | 164 |
| Bing | <https://www.livestrong.com/article/475904-the-best-foods-to-eat-if-you-have-sarcoidosis/> | 171 |
| Bing, Yahoo | <https://www.bumc.bu.edu/pulmonary/research/translational/sarcoidosis/> | 175 |
| Bing | <http://www.itmonline.org/arts/sarcoidosis.htm> | 176 |
| Bing, Yahoo | <https://house.fandom.com/wiki/Sarcoidosis> | 178 |
| Bing | <https://findanyanswer.com/can-sarcoidosis-affect-the-uterus> | 193 |
| Bing | <http://www.breathingmatters.co.uk/sarcoidosis/> | 195 |
| Bing | <https://www.health.com/condition/skin-conditions/what-is-sarcoidoisis> | 196 |
| Bing | <https://www.sukhayuayurved.com/pulmonology/ayurveda-treatment-for-respiratory-diseases/sarcoidosis/> | 198 |
| Yahoo | <https://www.urmc.rochester.edu/pulmonary/patient-care/sarcoidosis-program.aspx> | 43 |
| Yahoo | <https://www.lifewithpulmonaryfibrosis.com/understanding-pulmonary-fibrosis/conditions-pulmonary-fibrosis/sarcoidosis> | 53 |
| Yahoo | <https://www.drugs.com/cg/sarcoidosis.html> | 56 |
| Yahoo | <https://www.infobloom.com/what-is-liver-sarcoidosis.htm> | 57 |
| Yahoo | <https://www.empowher.com/community/ask/what-are-signs-flare-due-sacoidosis> | 58 |
| Yahoo | <https://muschealth.org/medical-services/lung-care/sarcoidosis> | 60 |
| Yahoo | <https://www.scientificamerican.com/article/experts-sarcoidosis-bernie-mac/> | 67 |
| Yahoo | <https://weiinstitute.org/sarcoidosis-treatment/> | 74 |
| Yahoo | <https://med.stanford.edu/sarcoidosis/about-.html> | 80 |
| Yahoo | <https://www.templehealth.org/services/lung/patient-care/programs/sarcoidosis> | 83 |
| Yahoo | <https://librepathology.org/wiki/Sarcoidosis> | 94 |
| Yahoo | <https://themighty.com/2016/09/what-living-with-sarcoidosis-feels-like/> | 97 |
| Yahoo | <https://health.ucsd.edu/specialties/pulmonary/advanced-lung-disease/Pages/sarcoidosis.aspx> | 99 |
| Yahoo | <https://byebyedoctor.com/sarcoidosis/> | 103 |
| Yahoo | <https://www.keefelaw.com/faqs/signs-and-symptoms-of-sarcoidosis-of-the-eyes.cfm> | 104 |
| Yahoo | <https://ufhealth.org/sarcoidosis> | 110 |
| Yahoo | <https://www.everydayhealth.com/sarcoidosis/guide/> | 113 |
| Yahoo | <https://improvised-home-remedies.com/how-to-treat-cure-sarcoidosis-naturally-at-home/> | 116 |
| Yahoo | <https://www.evenbetterhealth.com/symptoms_of_sarcoidosis.php> | 122 |
| Yahoo | <https://www.belmarrahealth.com/cardiac-sarcoidosis-causes-symptoms-treatment/> | 126 |
| Yahoo | <https://patient.info/forums/discuss/stage-1-2-3-4-in-sarcoidosis-what-does-this-mean--273475> | 128 |
| Yahoo | <https://acthar.com/sarcoidosis> | 131 |
| Yahoo | <https://www.upstate.edu/pulmcc/healthcare/subspecialty/sarcoidosis.php> | 133 |
| Yahoo | <https://medicine.uiowa.edu/iowaprotocols/parotid-sarcoidosis> | 134 |
| Yahoo | <http://peripheralneuropathycenter.uchicago.edu/learnaboutpn/typesofpn/inflammatory/sarcoidosis.shtml> | 143 |
| Yahoo | <https://sarcoidsoldier.wordpress.com/sarcoidosis/> | 146 |
| Yahoo | <https://www.epainassist.com/chest-pain/lungs/sarcoidosis-or-sarcoid> | 147 |
| Yahoo | <https://www.ssdrc.com/ssd-sarcoidosis.html> | 154 |
| Yahoo | <https://www.ladycarehealth.com/top-natural-cures-for-sarcoidosis/> | 157 |
| Yahoo | <https://askjan.org/disabilities/Sarcoidosis.cfm> | 169 |
| Yahoo | <https://www.hcplive.com/view/what-know-about-sarcoidosis-disease> | 175 |
| Yahoo | <https://www.leaf.tv/6144967/are-there-herbal-treatments-for-sarcoidosis/> | 187 |
| Yahoo | <https://www.aarda.org/diseaseinfo/sarcoidosis/> | 190 |
| Yahoo | <https://www.rxlist.com/sarcoidosis/definition.htm> | 195 |
| Yahoo | <https://www.ibenedictines.org/2015/10/24/living-with-sarcoidosis-and-sarcoma/> | 198 |

**Supplement S4** Characterization of unique websites by category.

HON, Health on the Net; JAMA, Journal of the American Medical Association; SD, standard deviation.

|  | | | **Academic/ Governmental organization** | **Foundation/ Advocacy** | **News/media** | **Industry/for profit organization** | **Personal commentary/ Blog** | **p value** |
| --- | --- | --- | --- | --- | --- | --- | --- | --- |
| **GENERAL INFORMATION** | **Websites, n (%)** | | 57 (46) | 16 (13) | 37 (30) | 9 (7) | 5 (4) |  |
|  | **Host continent,**  **n (%)** | Europe | 8 (42) | 4 (21) | 4 (21) | 1 (5) | 2 (11) |  |
|  |  | North America | 44 (46) | 11 (12) | 31 (33) | 6 (6) | 3 (3) |  |
|  |  | Asia | 0 (0) | 0 (0) | 1 (33) | 2 (67) | 0 |  |
|  |  | Autralia | 5 (71) | 1 (14) | 1 (14) | 0 | 0 |  |
|  | **Update** | Publishing/update date available, n (%) | 27 (42) | 6 (9) | 26 (41) | 1 (2) | 4 (6) |  |
|  |  | Time in months since upload/update, median (range) | 23 (0-323) | 17 (13-82) | 24 (0-260) | 102 (102) | 70 (1-84) |  |
|  |  | Time in months since upload/update, mean (SD) | 47 (70) | 28 (27) | 50 (72) | 102 (0) | 56 (38) |  |
| **GENERAL QUALITY OF INFORMATION ONLINE** | **HON foundation certificate** | Available/valid | 4 (7) | 0 (0) | 14 (38) | 0 (0) | 0 (0) |  |
|  |  | Not available/not valid | 53 (93) | 16 (100) | 23 (62) | 9 (100) | 5 (100) |  |
|  | **JAMA score** | Median (range) | 2 (1) | 2 (1) | 3 (1) | 1 (1) | 2 (1) | <0.001 |
